# Implementation barriers and enablers of midwifery group practice for vulnerable women: a qualitative study in a tertiary urban Australian health service

**DOI:** 10.1186/s12913-022-08633-8

**Published:** 2022-10-19

**Authors:** Patricia A Smith, Catherine Kilgour, Deann Rice, Leonie K Callaway, Elizabeth K Martin

**Affiliations:** 1 Women, Children and Families Stream Metro North Health, Butterfield Street, 4029 Herston, Brisbane, QLD, Australia; 2 Women’s and Newborn Services, Royal Brisbane and Women’s Hospital, Butterfield St, 4029 Herston, Brisbane, QLD, Australia; 3 School of Nursing, Midwifery and Social Work, The University of Queensland, St Lucia, 4072 Brisbane, QLD, Australia; 4 Faculty of Medicine, The University of Queensland, Herston Road, 4006 Herston, Brisbane, QLD, Australia; 5 Mater Research Institute, Faculty of Medicine, University of Queensland, Raymond Terrace, 4101 South Brisbane, Brisbane, QLD, Australia

**Keywords:** Midwifery, Midwifery Group Practice, Continuity, Pregnancy, Vulnerable Women, Implementation science

## Abstract

**Background:**

Maternity services have limited formalised guidance on planning new services such as midwifery group practice for vulnerable women, for example women with a history of substance abuse (alcohol, tobacco and other drugs), mental health challenges, complex social issues or other vulnerability. Continuity of care through midwifery group practice is mostly restricted to women with low-risk pregnancies and is not universally available to vulnerable women, despite evidence supporting benefits of this model of care for all women. The perception that midwifery group practice for vulnerable women is a high-risk model of care lacking in evidence may have in the past, thwarted implementation planning studies that seek to improve care for these women. We therefore aimed to identify the barriers and enablers that might impact the implementation of a midwifery group practice for vulnerable women.

**Methods:**

A qualitative context analysis using the Consolidated Framework for Implementation Research was conducted at a single-site tertiary health facility in Queensland, Australia. An interdisciplinary group of stakeholders from a purposeful sample of 31 people participated in semi-structured interviews. Data were analysed using manual and then Leximancer computer assisted methods. Themes were compared and mapped to the Framework.

**Results:**

Themes identified were the woman’s experience, midwifery workforce capabilities, identifying “gold standard care”, the interdisciplinary team and costs. Potential enablers of implementation included perceptions that the model facilitates a relationship of trust with vulnerable women, that clinical benefit outweighs cost and universal stakeholder acceptance. Potential barriers were: potential isolation of the interdisciplinary team, costs and the potential for vicarious trauma for midwives.

**Conclusion:**

There was recognition that the proposed model of care is supported by research and a view that clinical benefits will outweigh costs, however supervision and support is required for midwives to manage and limit vicarious trauma. An interdisciplinary team structure is also an essential component of the service design. Attention to these key themes, barriers and enablers will assist with identification of strategies to aid successful implementation. Australian maternity services can use our results to compare how the perceptions of local stakeholders might be similar or different to the results presented in this paper.

**Supplementary Information:**

The online version contains supplementary material available at 10.1186/s12913-022-08633-8.

## Background

Pregnant women with a history of substance use, mental health challenges and complex social issues have unique care needs during pregnancy, birth and the postpartum period [1] and are estimated to represent over 5% of the approximately 295,000 women who give birth each year in Australia [2]. There is strong international evidence that these women’s pregnancies are more likely to result in placental abruption, preterm birth, neonates that are small for gestational age, and neonatal admission to an intensive care unit [3, 4]. Vulnerable women may also experience domestic and family violence isolation in addition to poor maternal health, further compromising the fetus and neonate [1].

Existing maternity services may not meet the needs of vulnerable women during pregnancy, resulting in non-attendance of scheduled antenatal care and raising the risk of poor maternal and neonatal outcomes [5]. Whilst Australian data on non-attendance at antenatal appointments is limited, international evidence suggests antenatal care is not well accessed by vulnerable women. In the United Kingdom, smokers were significantly more likely to have a late booking appointment after 18 weeks gestation (Odds Ratio 1.6) [6]. In Finland, women who smoked and consumed alcohol were significantly more likely to receive insufficient antenatal care defined as between zero and five visits (Odds Ratios 1.87 and 1.48 respectively) [7]. Other studies in New Zealand, Belgium and the Netherlands demonstrated low antenatal care attendance by women with social and other vulnerability [8, 9]. Developing and implementing an antenatal, birth and postnatal service that addresses the challenges experienced by vulnerable women requires consideration of potential barriers to and enablers of successful care at both the health care provider and broader health service levels.

Continuity of care and carer is known to provide many benefits in terms of the health outcomes of mothers and babies and the levels of satisfaction of care for both consumers and care providers [10]. In Australia, most available and publicly funded models of maternity care are fragmented with limited continuity of carer across the whole pregnancy and post-partum period [11]. These Australian models facilitate monitoring of antenatal clinical indicators but may miss an opportunity to establish trusting relationships through continuity of carers during pregnancy and the postpartum period [12]. Establishment of trusting relationships is likely to improve attendance at care and enable discussion of behaviour changes during pregnancy. An alternative to this is Midwifery Group Practice (MGP), also called continuity of midwifery or caseload midwifery which has been implemented globally and largely focusses on low-risk pregnancies [10, 13]. However, such a model of care is rarely available for Australian women with complex social circumstances or with specific cultural or other needs [11]. The benefits of continuity of care and carer are well documented [10] and are likely to be seen in vulnerable women. Such antenatal care may facilitate equitable access, high quality health care and the best possible health outcomes during pregnancy, birth and the postpartum period [12].

Midwifery and midwifery group practice is recommended for all vulnerable women [12, 14–16] because of improved health outcomes for both mothers and babies. There are benefits in the areas of child protection interventions [17], mental illness [18, 19], substance-use [15, 20, 21] and infant neurodevelopment problems [22]. An absence of continuity of care has been identified as a barrier to seeking help for mental illness [18, 23]. Relationships between women and carers that are grounded in an interdisciplinary continuity of care and carer model increases women’s access to services and provide safe spaces for disclosure of sensitive information that guides high quality health care delivery [19]. High levels of consumer satisfaction are reported by women who experience midwifery group practice. Midwifery group practice is also safe in terms of maternal and neonatal outcomes, is associated with a reduction in maternal risk-taking behaviours, and is a less-costly model of maternity care [24–26]. While midwifery group practice for vulnerable women is recommended based on evidence from small international studies, it is not known how widespread or effective the model is in Australia.

It is also unknown how accepted midwifery group practice is amongst key stakeholders in Australia. Midwifery group practices have been established for Torres Strait Islander women and women carrying Aboriginal and Torres Strait Islander babies in recognition of the importance of continuity of carers for this vulnerable group [27, 28]. These midwifery group practices cater for only a small proportion of vulnerable pregnant women and so many other women continue to have antenatal care that does not meet their needs. One study has reported that health professionals are supportive of an interdisciplinary midwifery group practice model of care for vulnerable women where disciplines provide specialty support with the benefits of a continuity of care model [29]. Furthermore, to ensure sustained success, implementation of a midwifery group practice for vulnerable women requires careful planning using the principles of implementation science [30]. Risk management assessment indicates the need to identify likely barriers and enablers. Effective processes and strategies used to implement a midwifery group practice for vulnerable women depend on full engagement of stakeholders and a clear picture of the health service context [30, 31]. Not all stakeholders may be supportive and there may be less-visible aspects of the health service that may make sustained implementation of a midwifery group practice for vulnerable women difficult.

To identify these potential barriers and enablers, a context assessment was undertaken for a proposed midwifery group practice for vulnerable women at a single site tertiary maternity service in Queensland, Australia. Our intent is to identify and share the results of the context assessment which can be applied to other maternity services across Australia and demonstrate implementation science methods as an appropriate approach. Our research may expedite the implementation of such a model of care in other Australian maternity services.

## Methods

We aimed to identify the potential barriers and enablers for implementing a midwifery group practice for vulnerable women. To do this, we conducted a qualitative context assessment using the Consolidated Framework for Implementation Research (CFIR) [31–33]. The CFIR was chosen to guide the context assessment because the process required engagement with individuals and groups across multiple levels of the health service and external stakeholders. The framework has five domains that reflect key elements of a health service that need to be investigated before implementing change in an established interdisciplinary service. These are: “intervention characteristics” such as details of the new service being proposed; “outer setting” such as external influencing factors; “inner setting” such as unique aspects of the health service itself; “characteristics of individuals” who are involved in and/or exposed to the new service; and “process” such as ways of implementing and evaluating the new service [31]. The CFIR has demonstrated applicability to data collection, analysis and implementation within maternity settings [34, 35] and provided a practical framework to assess the multiple factors involved in planning for a new midwifery group practice for vulnerable women.

### Setting and participants

The 1000-bed health service is located in an inner suburb of an Australian capital city, with a diverse population catchment including large numbers of disadvantaged groups. There are 8000 staff on campus encompassing a multitude of complex health services [34] This study setting was selected as midwifery managers sought to improve accessibility of care delivered to vulnerable women, acutely aware of a high failure-to-attend rate where more than 25% (n = 205) of vulnerable women do not attend their scheduled antenatal care compared to 8% in the general population each year (unpublished health service data available on request). Whilst some MGP’s are already established there is no ability to be engaged concomitantly with the antenatal model of care available to vulnerable women [36]. As a specialty service the number of staff involved with the model of care for vulnerable women is small. In total, 40 internal and external stakeholders were invited to participate including: medical, nursing, midwifery, allied health, business, administration, and consumer representatives.

### Process

Qualitative semi-structured interviews (individual and small group) were designed using questions from the CFIR toolkit that explored each CFIR domain [32]. Some questions were adapted for local contextualisation and conversation style. Participant knowledge and awareness was assessed through the use of nine open-ended questions (Table 1). Participants (n = 9) in management / leadership roles were invited to respond to an additional question in relation to costs. A modified survey was completed by the business representative officer with clinically based questions removed and others added to retain a business and cost focus. As the interviews were guided by the CFIR, questions were not pilot tested.

**Table 1:** Collated interview questions mapped to Consolidated Framework for Implementation Research (CFIR) domains

CFIR Domain	Interview Question
Intervention Characteristics	What kind of information or evidence are you aware of that shows whether MGP would work in this setting?
	What advantages and disadvantages does MGP have for the needs for vulnerable women?
	What kinds of change would need to be made for an MGP to be effective for vulnerable women?
	What costs will be incurred to implement MGP for vulnerable women?
Outer Setting	How well do you think the women who would use the current service will respond to an MGP?
	Can you tell me what you know about other organisations that have implemented MGP or similar programs for vulnerable women? What kind of local, state or national performance measures, policies, regulations or guidelines influence the decision?
Inner Setting	What is the general level of knowledge and acceptance at the hospital for an MGP or similar program for vulnerable women?
	To what extent might the implementation take a back seat to other high priority initiatives going on now?
Characteristics of Individuals	How do you feel about an MGP for vulnerable women?
Process	To what extent has the organisation / unit set goals for implementation of further continuity of care models with a known midwife (through Midwifery Group Practice)? How important do you think it is to implement the intervention compared to the other priorities? How important is it to others, such as your co-workers or leaders, to implement the intervention compared to the other priorities? (Question for the business representative only).

The participants identified through purposeful sampling [37] were sent invitations including an information sheet providing a brief background to the study, via email, with open invitations also promoted at staff meetings. Participants could choose between attending individual or small group interviews that lasted 20 to 30 min. Thirteen individual interviews and 7 group sessions were held over a month’s period in 2019 on-site at the facility. Group interviews included between two and five members based on availability of attendees. All interviews were conducted in-person except for two where phone interviews were used at the request of the participants. Two female researchers (PS and DR) conducted the interviews with PS facilitating all, and DR co-leading all but three sessions due to her clinical availability. The two novice researchers, both dual registered nurses and midwives at senior and middle management levels were known to all participants. Demographic data were collected from all participants by a written survey at the interview to provide an overview of participants’ characteristics. Interviews were recorded with participant consent and transcribed verbatim by an administration support officer. Notes were also made by the interviewers. All data were de-identified in preparation for data analysis. Following the interviews, participants were notified that their de-identified information would be considered in subsequent planning for a midwifery group practice for vulnerable women. Participants were also invited to provide further information via email at their convenience, but transcripts would not be returned to comments for comment or correction. Information received by email post-interview was collated for de-identification and included in the analysis.

The study was deemed by the hospital’s and university’s Human Research Ethics Committees as a quality assurance or quality improvement activity and thus not requiring formal ethics approval (Exemption number: LNR/2019/QRBW/54,360). As part of this ethical review, we were required to provide brief background information on the proposed MGP model of care to participants. Background information included one sentence about the evidence for midwifery group practice being an appropriate solution for vulnerable pregnant women and the purpose of the study and risks and benefits of participating. Participants were also advised their involvement was voluntary and that their responses could be withdrawn at any stage up to two-weeks post-interview. Feedback on study results at completion of the study was provided to participants who requested follow-up.

### Data Analysis

In the initial manual thematic analysis, two midwife researchers used a grounded-theory approach and facilitated emergence of multiple themes from the data [38]. Each researcher independently analysed the data, highlighted key terms and assigned their own codes. The researchers conferred to agree on a joint understanding of the themes which emerged. Key words and phrases which were repeated amongst participants were tabulated. Agreed key words and phrases were organised into barriers and enablers as determined by consensus amongst the researchers and then coded “in vivo” using participants’ own words. As patterns emerged in the reassembling of data and coding, recurrent themes were identified to enable thematic analysis [38]. A coding tree was not created, as software was not used for this component of the analysis.

Reflexivity and reduction of potential researcher bias was identified and considered throughout the interview and analysis processes [38]. The midwife researchers reflected on and acknowledged both the potential bias of being midwives investigating a topic they may have a self-interest in, along with the benefits of improved engagement from participants as they were known colleagues.

To promote rigor and dependability in the study findings, a second round of analysis was conducted [39–41]. Peer checking was undertaken independently (by CK) through analysis of the de-identified research transcripts using Leximancer V4. Leximancer is a well-known text mining software used to identify concepts grounded in the study data [39] and has been used in Australian maternity settings [42, 43]. Peer checking using a text mining software reduces inadvertent researcher bias and provides another level of dependability within the study findings. The second analysis was compared with themes from first round analysis thus establishing findings across three researchers using two methods, and substantiating trustworthiness in the study [44]. If there were differences in manual and computer analysis results, the research team planned to reach a consensus on emerging themes through discussion. Themes were then mapped to CFIR domains and constructs within each domain to finalise the analysis. Key phrases and meaning from interview data were used to allocate themes to constructs.

## Results

Of the 40 people invited to an interview, 31 consented to participate (77.5%) with no response received from 9 others. No additional people contacted the study team to offer their involvement. Twenty individual or small group interviews were conducted. Data saturation was achieved following 10 interviews however, the research team elected to continue data collection due to many stakeholders wishing to participate. Most participants were female (87%) and between 41 and 50 years of age (35.5%). They were predominantly permanent employees (74.2%) and had more than 10 years’ experience (70.9%) (Table 2). Participant characteristics generally aligned with that of the Australian health workforce, with most participants being nurses and midwives (Australian Institute of Health and Welfare, 2020). However, more people aged 41 and over participated in the research, whereas the comparable Australian workforce is mostly aged 20 to 34 years.

**Table 2:** Characteristics of participants [N = 31]

Participant characteristic	n (%)
Age 20–30 31–40 41–50 51–60 61+	2 (6.5) 6 (19.4) 11 (35.5) 9 (29) 3 (9.6)
Gender Male Female Not stated	3 (9.7) 27 (87) 1 (3.2)
Stakeholder role Midwife Medical Officer Nurse Nurse/Midwife Leader Allied Health Practitioner Other (Business Representative, Administration Officer, Consumer)	13 (41.9) 5 (16.1) 6 (19.4) 3 (9.7) 1 (3.2) 3 (9.7)
Work hours Full time Part time Not stated	15 (48.4) 13 (41.9) 3 (9.7)
Employment status Temporary appointment Permanent appointment Not stated	1 (3.2) 23 (74.2) 7 (22.6)
Professional experience* < 5 years 5–10 years 10 + years Not stated	0 7 (22.6) 22 (70.9) 1 (6.5)

*The consumer representative was not included in this group, thus n = 30

Potential barriers and enablers were identified from the interview data and grouped into themes: the woman’s experience, midwifery workforce, gold standard care, the interdisciplinary team, and costs. These themes function as ways in which we have organised and expressed the barriers and enablers. A summary of potential barriers and enablers from which these themes emerged is presented in Supplementary File 1.

### The woman’s experience

Discussion around the woman’s experience identified enablers for the proposed model. Many participants expressed empathy for the likely patient cohort and placed the proposed model as a high priority for the health service. Enablers included perceptions of improved clinical outcomes, building of safety and trust, patient satisfaction and the potential to reduce ‘fail to attend’ rates at the specialty clinic. This is supported by comments from participants:

*… vulnerable or disadvantaged groups would benefit …* (Other role, Interview 1).

*… some with abuse histories don’t want to go over those histories over and over*

*again…some come with diverse cultural situations and specific needs …* (Midwife, Interview 2).

… *they can build up trust, they can have the tough talk with a familiar face …* (Nurse, Interview 12).

The general sentiment expressed by many participants is captured by this statement:

*…the patient can build a rapport and have trust with the clinicians…. It not only makes the patient feel safe and more comfortable with their surroundings, in my opinion, it makes the patient a bit more accountable and builds rapport with that clinician and women would be more engaged to come back…* (Other role, Interview 1).

The proposed model was being viewed as positive and with a woman-centred focus. These views become potential enablers when preparing detailed business cases that demonstrate strategic and strong stakeholder support as well as alignment with evidence and policy advocating woman-centred care.

However, potential barriers centred around concerns that women may disengage if they did not bond with the known midwife, were socially isolated or feared being reported to child safety services:

*For the women it’s positive all around, unless they felt they couldn’t engage with the midwife, which could lead to the women disengaging completely.* (Midwife, Interview 6)

*The women can be fearful about opening up…that there may be negative consequences…* (Midwife, Interview 10).

Participants also suggested ways to address the woman’s disengagement through awareness and actions by the midwives:

*We have to be open and transparent…so there’s a consistent approach and that we can be strong and recognise it when it (disengagement) happens and maintain the relationship …* (Midwife, Interview 10).

*… the midwife would need to be capable of referring… to those that can help …* (Midwife, Interview 16).

*… if that relationship isn’t working there could be space to swap …* (Other role, Interview 17).

While women’s disengagement from the proposed model might be identified as a risk, in discussions around this barrier solutions were identified. Such solutions would inform risk mitigation.

### Midwifery workforce

Participants saw the proposed new model as an opportunity for midwives to gain new skills and expand their scope of practice which was identified as an enabler:

*There are a few midwives out there currently upskilling themselves and are really passionate and interested and already preparing for being part of the team …* (Nurse/Midwife Leader, Interview 3).

*Midwives will actually see this as a positive move and it’s going to be development for them, and it’s going to be opportunistic for them …* (Nurse/Midwife Leader, Interview 4).

A major workforce barrier to implementation success was how midwifery group practice would place high psychological demand on the midwives leading to burnout or vicarious trauma:

*… very complex women with personality disorders and high psychological needs and that could be quite demanding of one midwife as the primary caregiver …* (Nurse/Midwife Leader, Interview 3).

*… to field all those phone calls and constantly support that person would be really challenging …* (Nurse/Midwife Leader, Interview 3).

*For the midwife dealing with only these women, it could over time be mentally challenging … potentially exhausting and tiring …* (Midwife, Interview 6).

The difficulty in attracting midwives to the proposed model of care was an identified barrier to implementing a high standard of care:

*… attracting the midwives that would have an interest in it, we almost need a mother-like figure…would have to be resilient and have had a few more life experiences …* (Medical Officer, Interview 13).

While this second barrier is contradictory to the initial workforce enabler identified in this theme, participants became solution focussed in the interviews, which is reflected in the theme reported below ‘The interdisciplinary team’.

### Gold standard care

The belief amongst participants of strong, quality evidence in favour of the proposed model was identified as an enabler, and there were no barriers highlighted by participants in terms of available evidence. Many participants identified through discussion around evidence that a midwifery group practice for vulnerable women would be a positive step for the women involved. This was particularly evident for participants who had undertaken reading prior to the interview with participants expressing:

*I have looked online to have a look at the research… shows the best outcome for babies and mothers across the board in terms of continuity of care models …* (Other role, Interview 1).

*MGP is gold standard and… there’s lots of research out there that shows that continuity of care is best for these women to develop a relationship …* (Midwife, Interview 2).

*… the people caring for them are more likely to pick up on deviations from a normal emotional state …* (Midwife, Interview 2).

Some participants had undertaken self-directed reading to source additional information regarding midwifery group practice and the needs of vulnerable women. Whilst participants were sent relevant information containing brief background to the proposed study in advance of the interview, some attendees advised they wanted to come well prepared. For example, two participants advised the researchers that they were very grateful to have been invited to interview and were now more aware of the benefits of midwifery group practice for women including that this care was “gold standard”. The integral way in which having a known midwife provides benefit and support for women was repeatedly discussed by participants. For many, the perception from the evidence that a midwifery group practice for vulnerable women is “gold standard” was the sole driver for supporting the proposed model and was seen as a strong enabler which would enhance a business case for the proposed model.

### The interdisciplinary team

Support for the inclusion of an interdisciplinary team in the proposed model of care was identified as an enabler and was particularly supported by non-midwife participants:

… *I would love it, because we felt [from observation of previous staffing arrangements] that when the midwives were staying in the role longer it felt very organised and that we knew the patients very well …* (Allied Health Practitioner, Interview 9).

*There’s also the opportunity to develop an interdisciplinary trust …* (Nurse, Interview 14).

Midwife participants felt similarly:

*Need a multi-disciplinary team …* and.

*“… still having that multi-disciplinary approach… is fantastic …* (Midwife, Interview 11).

Other responses emphasised the importance of midwives working with other disciplines and not practising in isolation:

*It’s really beneficial having a whole team caring for them* (Midwife, Interview 2) *…a*nd.

*… continuing to have the multi-disciplinary input and multi-disciplinary team meeting is going to be beneficial …* (Medical Officer, Interview 20).

Others commented:

*… it’s an addition to the multi-disciplinary teams, so it doesn’t take anything away but they’ve got some-one they can trust that follows them through all the way …* (Medical Officer, Interview 8).

*… the midwives … would need to be engaged with the multi-disciplinary team more than … MGP’s …* (Other role, Interview 17).

The overall sentiment across disciplines was that:

*Everyone’s ready for a change in the space and a growth in the space and how we can improve for the women and I think it would be highly supported, valued and everyone would be on board …* (Nurse/Midwife Leader, Interview 3).

An interdisciplinary team was therefore an important and well supported component of the model, and no barriers to including an inter-disciplinary team in the model were identified. Participants were clear that midwives would lead continuity of care, while having expert health professionals involved to provide comprehensive care for the women. They would also provide professional support for midwives who might feel isolated in their role. These design details can be included in a business case to ensure successful and sustained implementation.

### Costs

Participants believed that the health benefits of the proposed model of care would outweigh the perception that a midwifery group practice for vulnerable women was a more expensive model of care. This belief was a clear enabler of the proposed model:

*… the cost would come with great reward …* (Nurse/Midwife Leader, Interview 3).

*… there will be extra cost, but the trade off in terms of good follow up might not save money but it will be money well spent …* (Medical Officer, Interview 8).

However, participants did express concern at the perceived increased need for resources and challenges with attributing costs to a range of clinical areas. The concern focussed around the potential impact on other teams which was seen as a barrier:

*I think there is resistance…they see this is going to be taking away from their skill mix and FTE (Full Time Equivalent) …* (Nurse/Midwife Leader, Interview 4).

However, other participants believed that these perceptions and challenges could be overcome and that the proposed model of care should be a priority for the hospital:

*… yes there are financial implications and barriers, there doesn’t seem to be good evidence to show why not …* (Other role, Interview 1).

*… yes, it is very important that we generate the activity to get something for the work that we’re doing, but at the end of the day we are looking at patient centred care, so if it’s easier and the best outcome for mother and baby… then that’s what we have to do …* (Other role, Interview 1).

*Why shouldn’t they have an MGP, they shouldn’t be excluded just because they’ve had drug and alcohol or mental health issues in the past …* (Nurse, Interview 15).

*I see it as high priority to look at how we can increase activity and treat this vulnerable group …* (Nurse/Midwife Leader, Interview 4).

Perceptions around cost that are both potential enablers and barriers to gaining support and successful implementation would need to be clarified as fact in a business case before the proposed model is implemented. Participants had concerns around costs which may be reflected by decision-makers when examining the rigor in which a business case has been prepared. This may also influence future expansion of midwifery group practice as a model of care more generally.

**Fig. 1:**
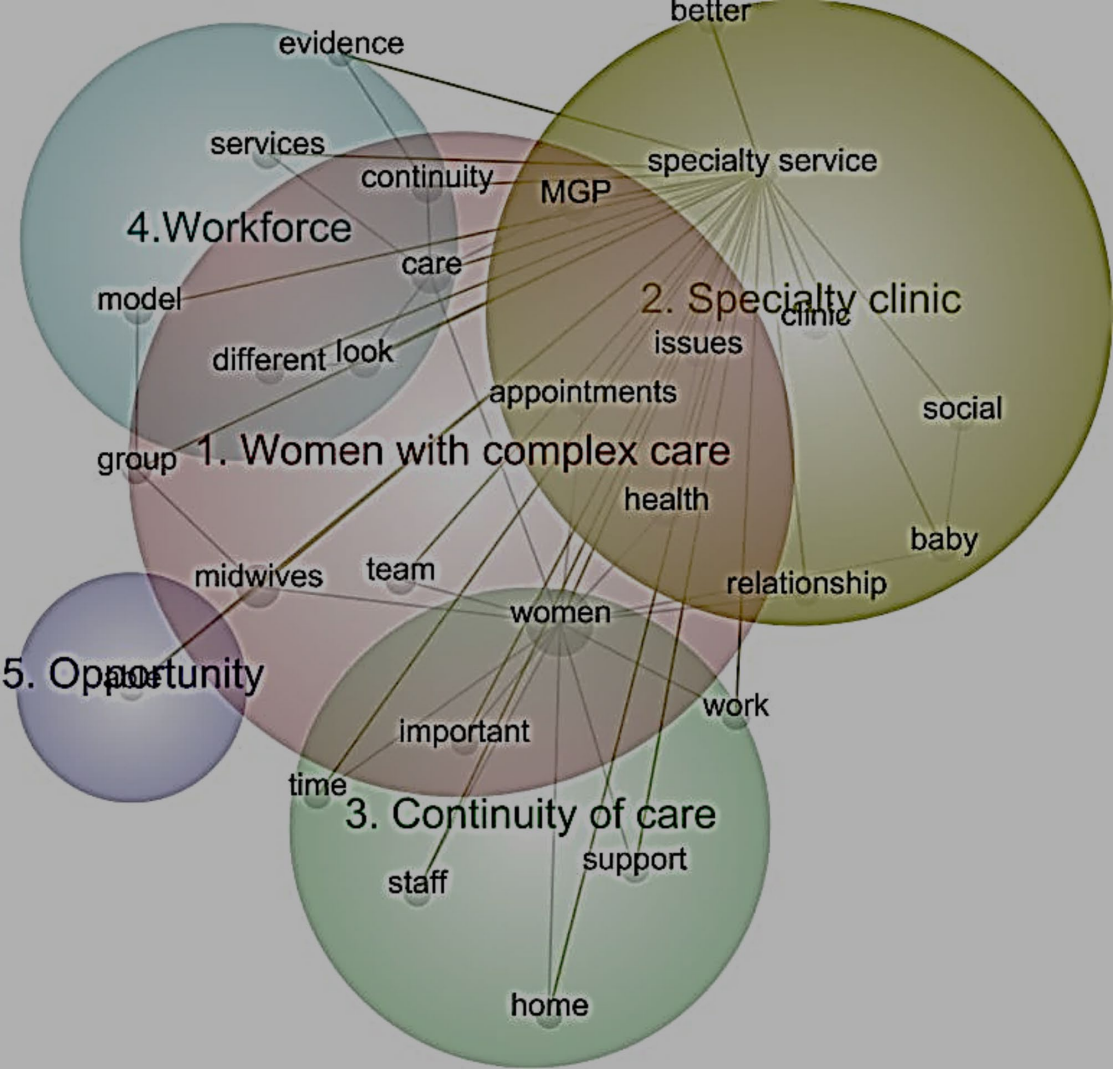
Leximancer 4™ generated themes from stakeholder interviews

In the computer-assisted analysis, five themes were identified from the data: women with complex care: specialty clinic; continuity of care; workforce; and opportunity (Fig. 1). Themes and sub-themes similar to those that emerged from manual analysis were around the woman’s experience, workforce, standards of care and opportunity.

Table 3 shows the mapped overarching themes and how they relate to the five CFIR domains and constructs. In this table it becomes clear what the requirements for successful and sustained implementation of the proposed model might be. The theme *Gold standard care* mapped to five constructs indicates that either a perception, or knowledge of evidence supporting midwifery group practice for vulnerable women would be critical to its success. A thorough consideration of potential experiences for the woman, the workforce and costs when preparing a business case, will be a determinant of model implementation success. An interdisciplinary team that is already part of the organisational structure and engaged in planning the model is likely to be essential. Processes that demonstrate evidence of planning and reflecting across all CFIR domains, especially regarding linkages between different health professional disciplines and costs, are also important.

**Table 3:** Summary of interview themes mapped to Consolidated Framework for Implementation Research (CFIR) domains and constructs

CFIR domains and constructs	Themes
**Intervention characteristics**	
Evidence strength and quality	Gold standard care
Relative advantage	The woman’s experience Midwifery workforce Costs
Cost	Costs
Adaptability	Midwifery workforce
**Outer setting**	
Patient needs and resources	The woman’s experience
Peer Pressure	Gold standard care
**Inner setting**	
Structural characteristics	The interdisciplinary team
Implementation climate	Midwifery workforce
Relative priorities	Gold standard care The woman’s experience
**Characteristics of individuals**	
Knowledge and beliefs about the intervention	Gold standard care
Individual stage of change	Gold standard care
Self-efficacy	Midwifery workforce
**Process**	
Planning	The interdisciplinary team
Reflecting and evaluating	Costs

## Discussion

In this study we have identified, with an interdisciplinary stakeholder perspective, both the potential barriers and enablers that will need to be considered in the next phases of planning and implementing a midwifery group practice for vulnerable women. Participants identified that the proposed model is likely to provide health benefits for women due to the rapport built with a small group of care givers including a supportive interdisciplinary team providing continuity of care. However, participants were conscious of the burden of such a maternity care model on the workforce, both in terms of the emotional challenge due to the women’s complex care requirements and managing the financial cost of the service which would require further evaluation.

When mapping the themes to the CFIR domains, the implications of local results to Australian maternity services became evident. Organising the data in this way also enabled understanding of factors that may lead to successful and sustained implementation of a MGP for vulnerable women internationally as the CFIR has become a universal implementation ‘language’.

### Intervention characteristics

Stakeholders perceived that there was high quality and valid evidence supporting midwifery group practice for vulnerable women as “*gold standard care*” (Midwife, Interview 2). Quality evidence drawn from Australian studies [10, 13] builds confidence amongst stakeholders, which is a strong enabler. Participants weighed the relative advantages of the proposed model of care over the existing care provided to vulnerable women and believed that the health benefits for the women and infants would outweigh the costs. This belief in the net benefit when costs were also considered was not rooted in available evidence, which contrasts with the desire for evidence for “gold standard” effectiveness. The cost of the proposed model had not been established but was imagined by participants to be higher than both the current model of care and comparative midwifery group practices.

Participants were mostly clinicians with more than 10 years’ experience and so were likely able to make accurate assumptions around the number of workforce hours required to build rapport with women, discuss the care being delivered with the women and amongst colleagues, and deliver the volume of care required to optimise health outcomes. However, there is no known evidence for the cost-effectiveness of such a niche model of care, only generalised costs reported for Australian midwifery group practice [10, 45]. For Australian maternity services looking to implement a midwifery group practice for vulnerable women, careful understanding of costs and transparent communication to decision-makers is needed. Our research suggests that stakeholders naturally seek evidence for both costs and effectiveness, and in the absence of evidence, local costs should be examined.

### Outer setting

A midwifery group practice was perceived to meet the needs of vulnerable women because for example, *“some with abuse histories don’t want to go over those histories over and over…”* (Midwife, Interview 2). The continuity of midwives would ensure the deep needs of each woman were met. However, concerns were expressed regarding when having a known midwife might be a disincentive for women to engage. Primarily this related to women with involvement of child protection services or times where personal factors impacted on building rapport and a therapeutic relationship was not established between the woman and the midwife. A midwifery group practice for vulnerable women should be designed with flexibility in the case where a rapport is not being established between the care givers and the woman [46]. The service design would need to ensure that midwives can be changed across groups.

### Inner setting

The structural characteristics of the setting for this research were that it is a mature and large maternity service, with a range of existing and well-supported midwifery group practices already established. Consequently, there may have been fewer inner setting barriers to establishing the proposed model of care compared to Australian maternity services in which midwifery group practice is new or not yet established. Further, the interdisciplinary team engaged in this research was supportive of midwifery group practice. Although, it was highlighted that any potential changes to how the team works with the midwifery group practice would need to be implemented carefully. The interdisciplinary team expressed views that to continue safe and effective care, it was important the team’s role be maintained. The implementation climate of the inner setting was generally supportive, and there were some important reflections from the interdisciplinary team that would need to be acknowledged when preparing a business case for the proposed model.

### Characteristics of individuals

Stakeholders had a positive attitude towards the intervention; they placed a high value on the proposed model of care. Participants even sought evidence in preparation for the interviews and ensured they were familiar with the proposal. This demonstrates that individuals’ knowledge and beliefs about the intervention was a strong enabler for proceeding with the proposal. Further, that demonstrated engagement in the context assessment by stakeholders was an indicator that individuals were at an advanced stage of change in relation to redesigning maternity care for vulnerable women. The identification of this enabler suggests that the initial enthusiasm for the intervention would sustain its implementation over time. The results also demonstrated a moderate level of self-efficacy – there were mixed beliefs amongst individuals in their own capabilities to deliver the model of care, while also identifying that the proposed model of care would provide an opportunity for midwives to build their self-efficacy through gaining new skills and expanding their scope of practice. Modifications would need to be made to traditional midwifery group practice design due to perceived heavy demands from deep engagement with vulnerable women and the potential for vicarious trauma, burnout, and other emotional impacts for the midwives. Participants suggested that to overcome this barrier, caseloads should be reduced, and midwives should be well supervised and mentored as described also by others [46], especially when caring for vulnerable women [47]. To enable such an intervention to be implemented in other Australian maternity services, stakeholders would need to have confidence in their ability to seek and interpret the evidence and have an awareness of the strengths and limitations of the workforce capabilities to execute the proposed model of care.

### Process

Conducting the context assessment instilled confidence in readiness of the maternity service to adopt the proposed change. The context assessment was conducted early in the planning stages of the intervention and the strong engagement suggested interest in the intervention and acceptance of the planning methods. This enabler resulted in positive and open communication and was an unintended consequence of the context assessment, as participation from a large and broad range of disciplines was not expected.

### Strengths

A strength of this study was the use of the CFIR to guide interviews, along with two independent forms of data analysis and comparison of study findings. The CFIR outlines domains and constructs that are associated with effective implementation of new interventions. Using the CFIR in this study presents results that may guide other Australian maternity services on how to best plan and implement a midwifery group practice for vulnerable women. An additional strength was the alignment of the manual and computer-assisted thematic results. Computer-assisted analysis was undertaken to mitigate the recognised and acknowledged potential inherent bias in qualitative analysis [41]. Independent analysis and consistency of results further enhances the credibility and trustworthiness of the study, along with research reflexivity throughout the study. The midwife researchers (PS and DR) concluded in their reflections that the broad range of disciplines from which participants were drawn resulted in very positive engagement from the team and enhanced marketing of the proposed change in service delivery. The diversity and large relative number of stakeholders involved in the study also ensured the qualitative data were reflective of a comprehensive sample from which data saturation was readily achieved.

### Limitations

A limitation of the study was that due to local facility arrangements for selection of consumer representatives only one consumer participated in the interviews. The perspectives of consumers, and staff who were less experienced and/or from culturally and socially diverse backgrounds were not given, nor was there discussion in the interviews about the impact of the proposed model on these women. In addition, the homogenous sample, high level of experience and mature age of participants may indicate unintended sampling bias. This may be further exacerbated by the reading and preparation prior to interview done by some participants. Active pursuit of the voices of consumers and staff from a representative range of backgrounds in planning new models of maternity care across Australia is recommended by the researchers. As this study was carried out in a facility with established and supported midwifery group practices, caution should be applied in generalising the specific local results to other services for which midwifery group practice is a new concept. Instead, our interpretation of results for Australian maternity services should prompt services to identify which of our results mapped to the CFIR domains are relevant, and how they might be similar or different to the local context.

## Conclusion

In this study, we have used the CFIR to identify potential barriers and enablers to implementing a midwifery group practice for vulnerable women, with both local and national relevance. There was recognition that the proposed model of care is supported by research and a view that clinical benefits will outweigh costs, however supervision and support is required for midwives to manage and limit vicarious trauma. An interdisciplinary team supporting the midwives is also an essential component of the service design. For maternity services seeking to implement a midwifery group practice for vulnerable women, our results can be leveraged to further investigate other local contexts, and quickly identify strategies for effective and sustained implementation of the new model of care [48].

## Electronic supplementary material

Below is the link to the electronic supplementary material.

Supplementary Material 1

## Data Availability

The dataset supporting the conclusions of this article are available from the corresponding author on reasonable request.

## List of abbreviations

CFIR: Consolidated Framework for Implementation Research
MGP: Midwifery Group Practice
