# Supplementary material for: Implementation barriers and enablers of midwifery group practice for vulnerable women: a qualitative study in a tertiary urban Australian health service
Source: BMC Health Serv Res. 2022 Oct 19;22:1265. doi: 10.1186/s12913-022-08633-8 (PMC9583548; doi:10.1186/s12913-022-08633-8)
Supplement: Supplementary file 1 — Supplementary Material 1 [file 12913_2022_8633_MOESM1_ESM.docx]

**Supplementary File 1**

Summary of Barriers and Enablers

| Domain | Summary of Barriers | Summary of Enablers |
| --- | --- | --- |
| 1.Intervention Characteristics | - Lack of awareness of evidence (8) - Lack of flexibility due to midwives being on call (8) - Transport /social isolation / itinerant / low socioeconomic status preventing access for women (7) - No consistent person (case manager) in clinic to answer calls and needs of women who just ‘show up’ (4) - Burn out / vicarious trauma of midwives (19) - Resources /scope of practice required to build skills of midwives (18) - Increased need for admin support - Difficulty managing many interdisciplinary team appointments (2) - Lack of physical location for MGP team (2) - Not bonding with known midwife (6) - Lower caseload number thus higher costs (3) - Professional boundaries – midwives becoming too close (3) - High FTA rate (5) - Sick leave/annual leave within a small team difficult to replace (3) - Stigma attached to this cohort of women (3) - Demanding cohort and caseload - Increased length of stay (12) - Disempower women - Safety for home visits (8) - Vehicle, phones, pagers, increase costs (4) - Movement of workforce (taking from other areas) (8) - Dedicated social worker (3) - Variable collaboration between midwives and obstetricians (3) - Too many complex issues to manage in one MGP - Require reduced caseload allocation (5) - Increased cost of 2 x midwife home visits (4) - Funded as a medical model for any revenue raising activity - Case Manager needed to work with MGP (4) - Too many women to fit into an MGP (2). | - No good evidence to show it’s NOT a good idea - Research to suggest its “Gold Standard” (7) - Everyone is talking about it (2) - Aligns with similar programs elsewhere (9) - Evidence for positive outcomes for women and babies (11). Outweigh costs (6) - Other MGPs are working well (3) - Flexibility of scheduling of appointments 6) - Reduced FTA (11) - Build relationships (safety & trust) with known midwife (28) - Ownership of each patient and their journey - More likely to share all the intimate details involved in their complex social history / avoid repeating (7) - Increase in health literacy / navigation (6) - Potential to decrease child safety involvement - Interdisciplinary team involvement (11) - Safety net for vulnerable women to contact 24/7 (2) - Non-judgmental (5) - High level of patient satisfaction (1) - Improved referrals to child health and other post-natal services and discharge information - Postnatal off site/ home visits and care included. - Alternate off-site location for appointments - Introduction of case manager as well as MGP (3) - Upskilling opportunity for midwives - Reflective supervision for midwives - Maintain mother / baby dyad. |
| 2.Outer Setting | - Lack of awareness of other facilities and similar models of care (10) - Too many MGPs – staff may be confused regarding what each MGP is for - Some obstetricians may not be supportive. - May not be appealing for women with child safety issues who don’t want authorities to be aware (5) - Lack of understanding of what an MGP is (for women) - Lack of flexibility with appointments (3) - Women may not want follow-up in their homes. | - Positive for women (20) - Improve attendance (9) - Women are currently requesting care by known midwife - Continuity of carer - Ability to see all disciplines in one day - Similar care as elsewhere (9) - Has a very flexible approach to appointments with suits women (7) - No-like service available in the district (16). |
| 3.Inner Setting | - Concerns of exclusion of the interdisciplinary team (5) - Burnout rate for Midwives (14) - Safety issues for home visiting (3) - Lack of suitably qualified Midwives (3) - Prejudice against the women (4) - Lack of understanding of how MGP operates (3). - Bias to why MGP for this cohort and not others. - Resistance from Unit Managers fearing loss of staff / FTE to MGP (3) - Experienced midwives in MGP but removing this experience from other areas (3). - Perceived inequitable workloads (3). - Lack of regulations or performance measures. | - Support for MGP’s in general (16) - Midwives already expressing interest in working with these vulnerable women - Awareness that MGP would meet needs of women and babies – high priority (11) - Midwives very supportive (2) - Gold Standard – what we should work towards. |
| 4.Characteristics of Individuals | Nil identified. | - Extremely supportive (16) - MGP Gold Standard (2) - Great benefit for the women (7) - Satisfying as a clinician to follow entire journey. |
| 5.Process | Nil specifically identified. | Nil specifically identified. |

*Note:* summary of data obtained from typed transcripts of interviews. FTE = full time equivalent. Numbers in brackets indicate the total number of participants who described this concept.
